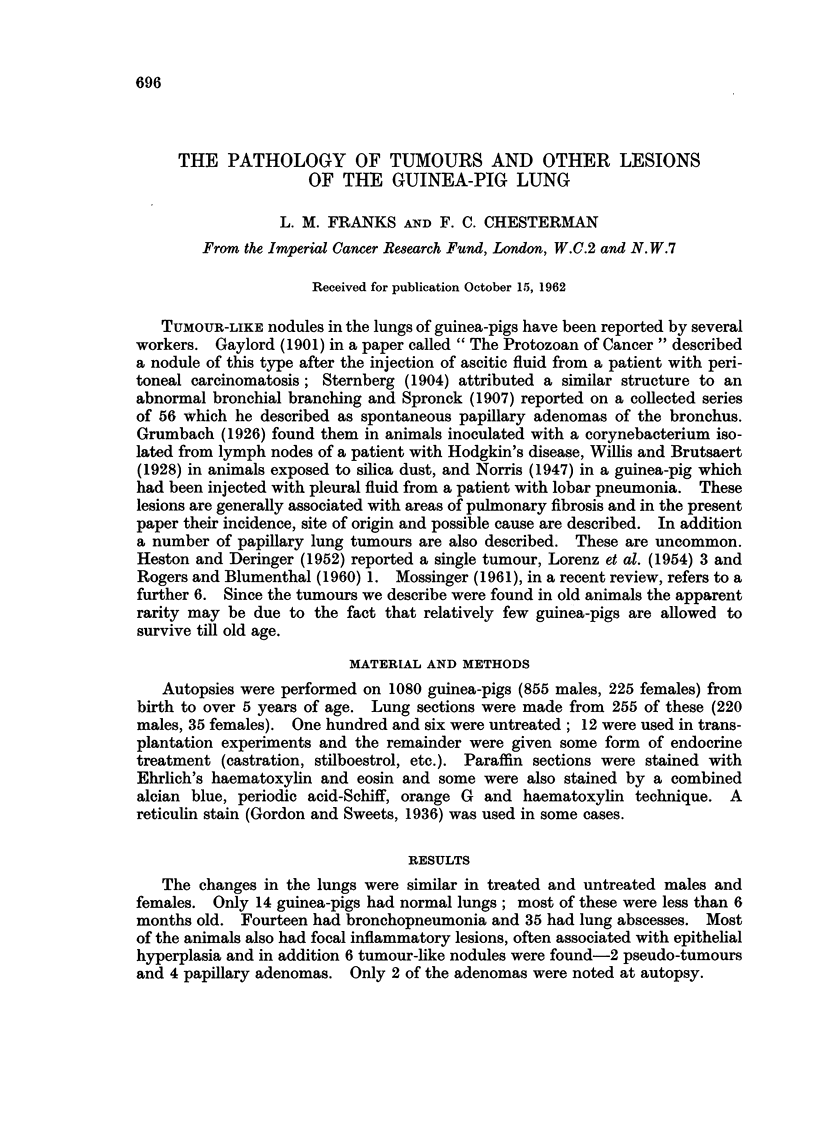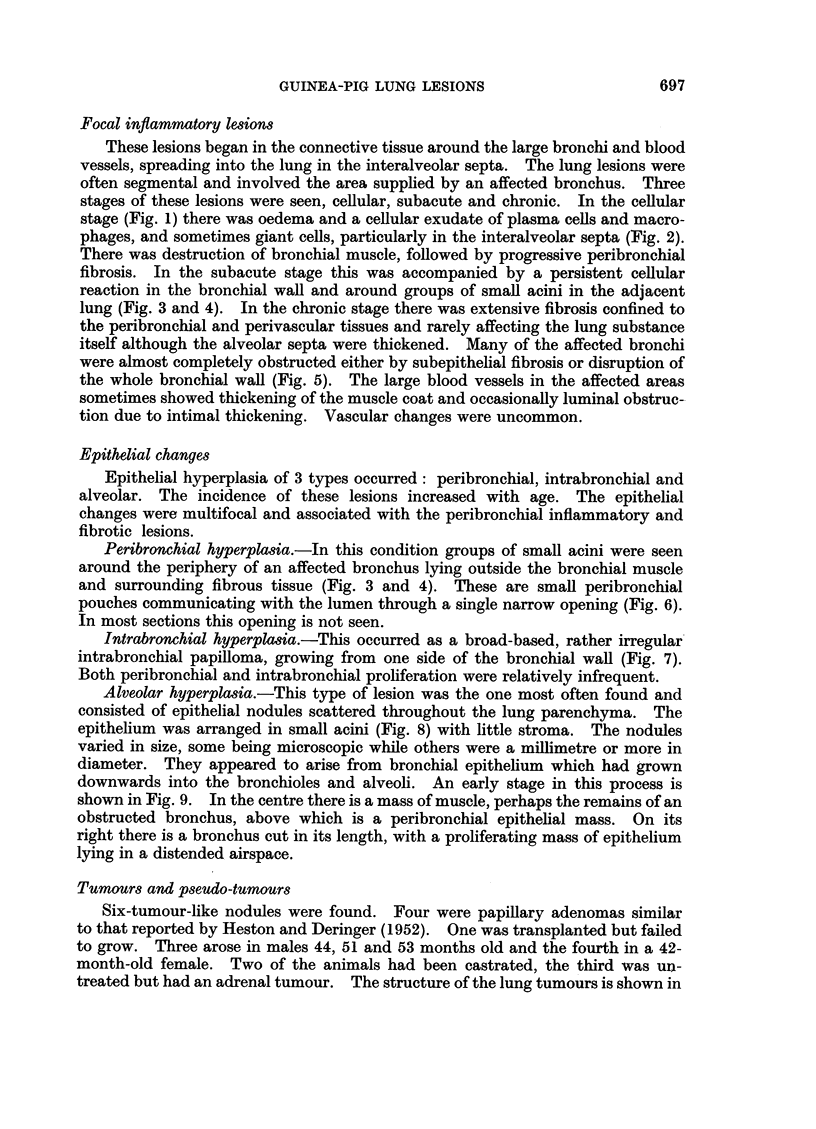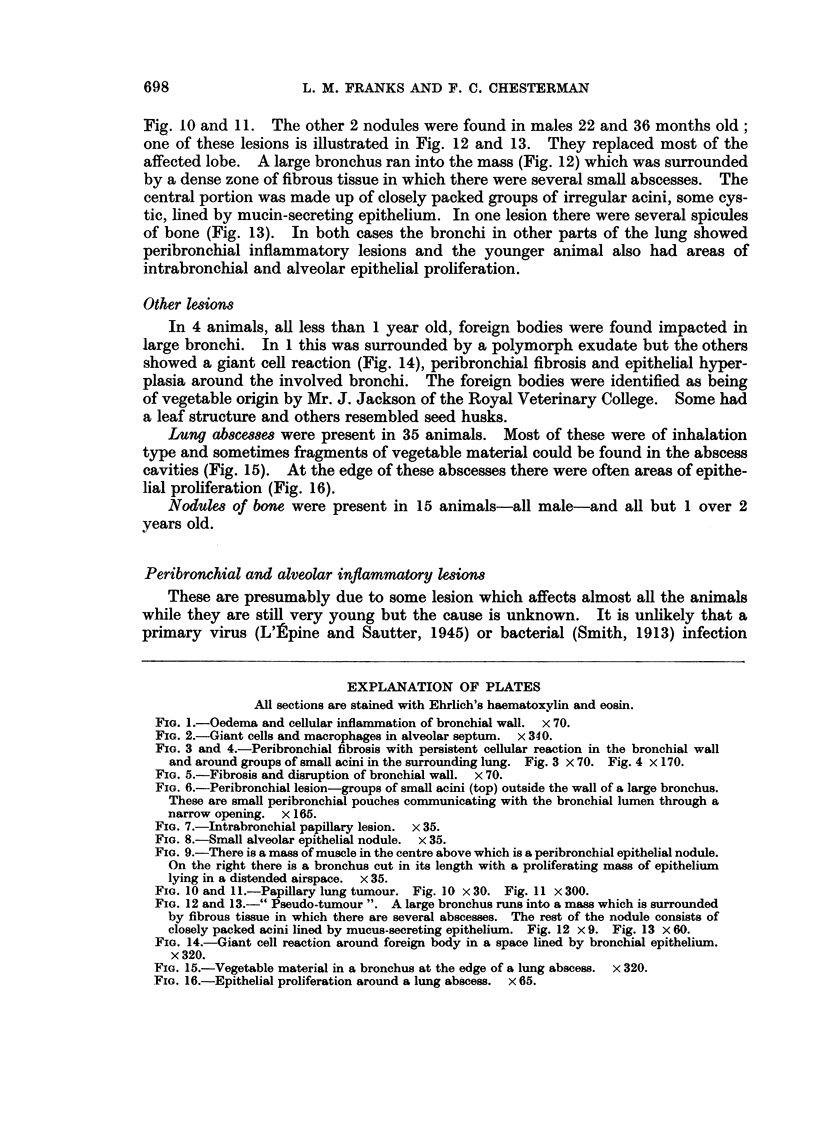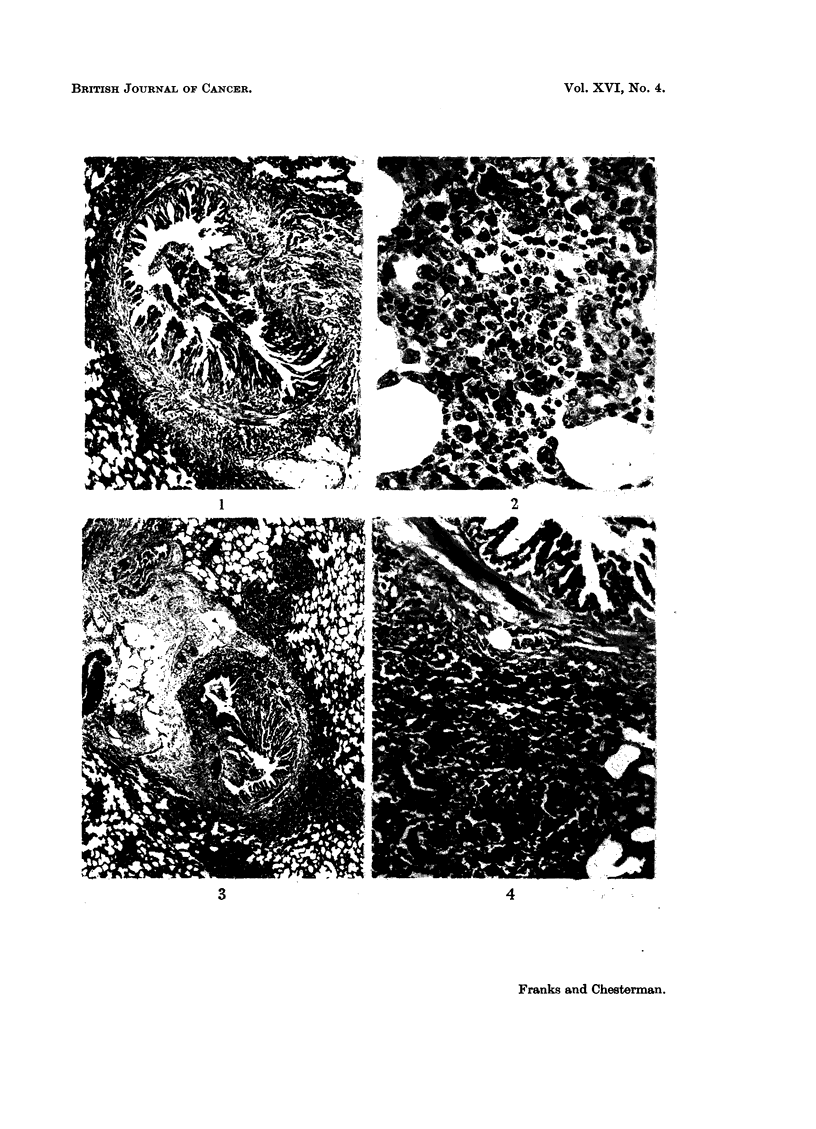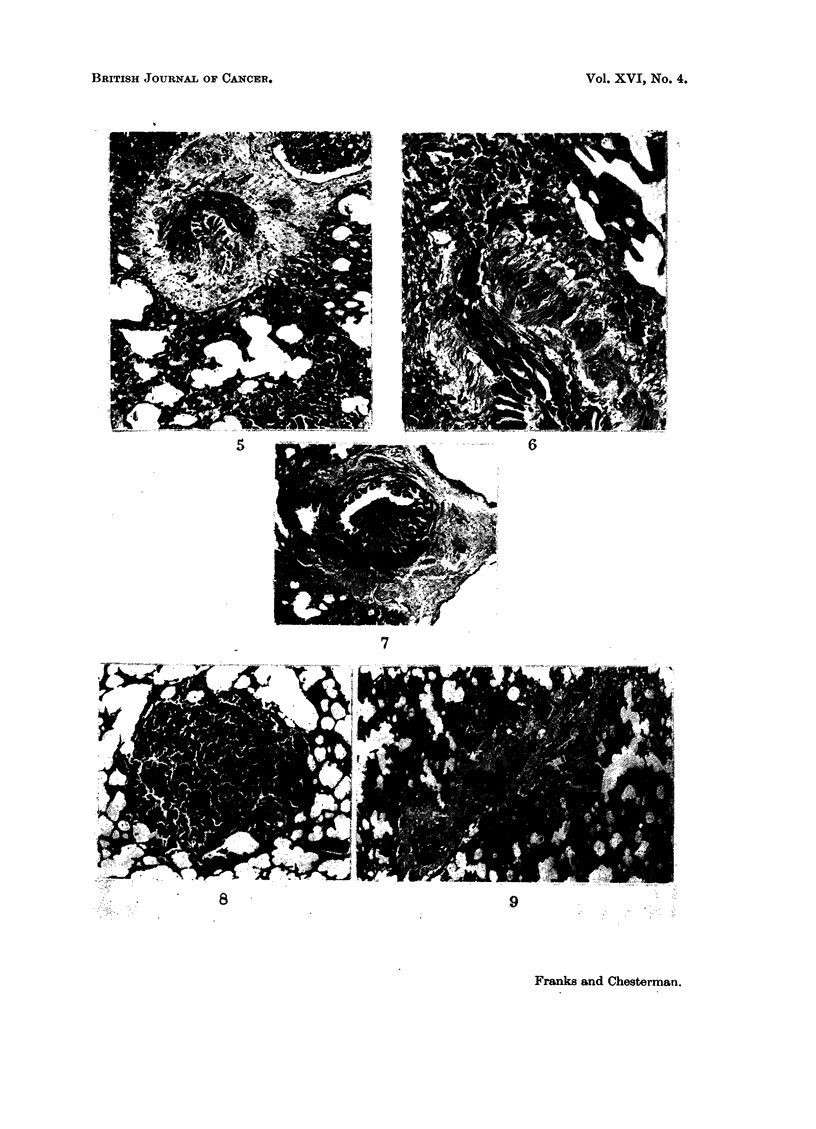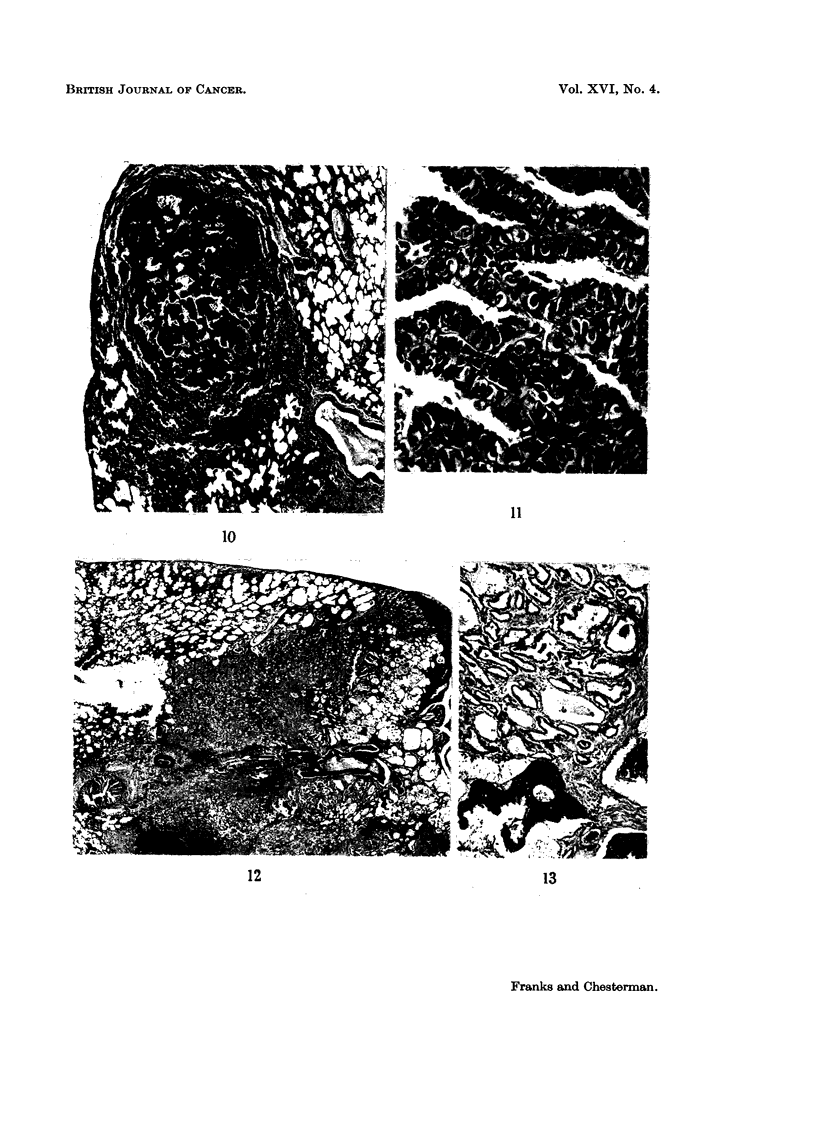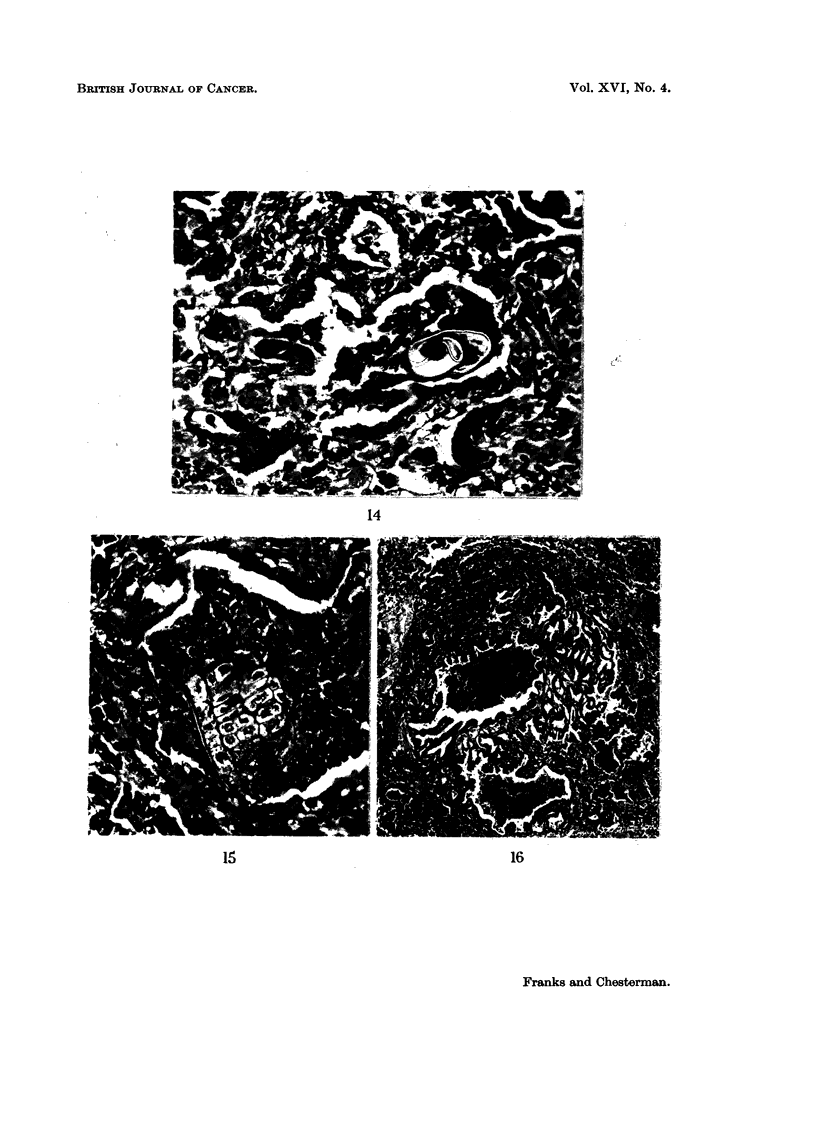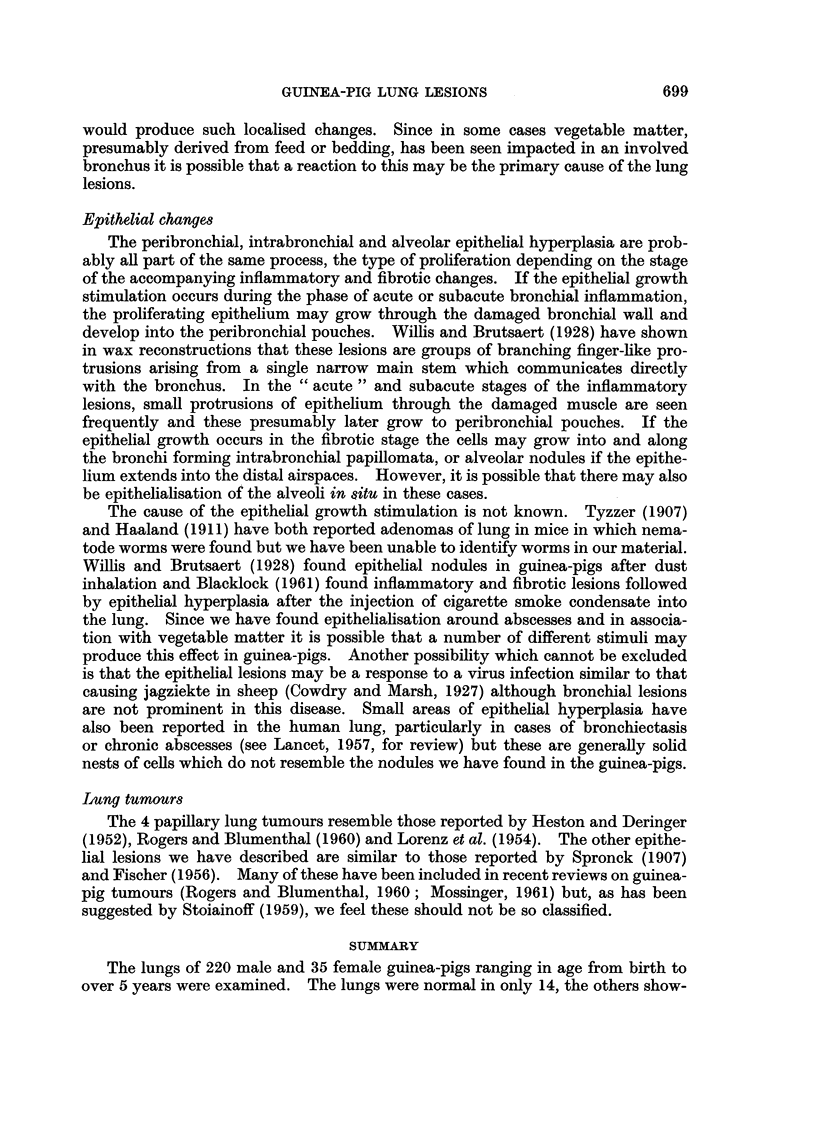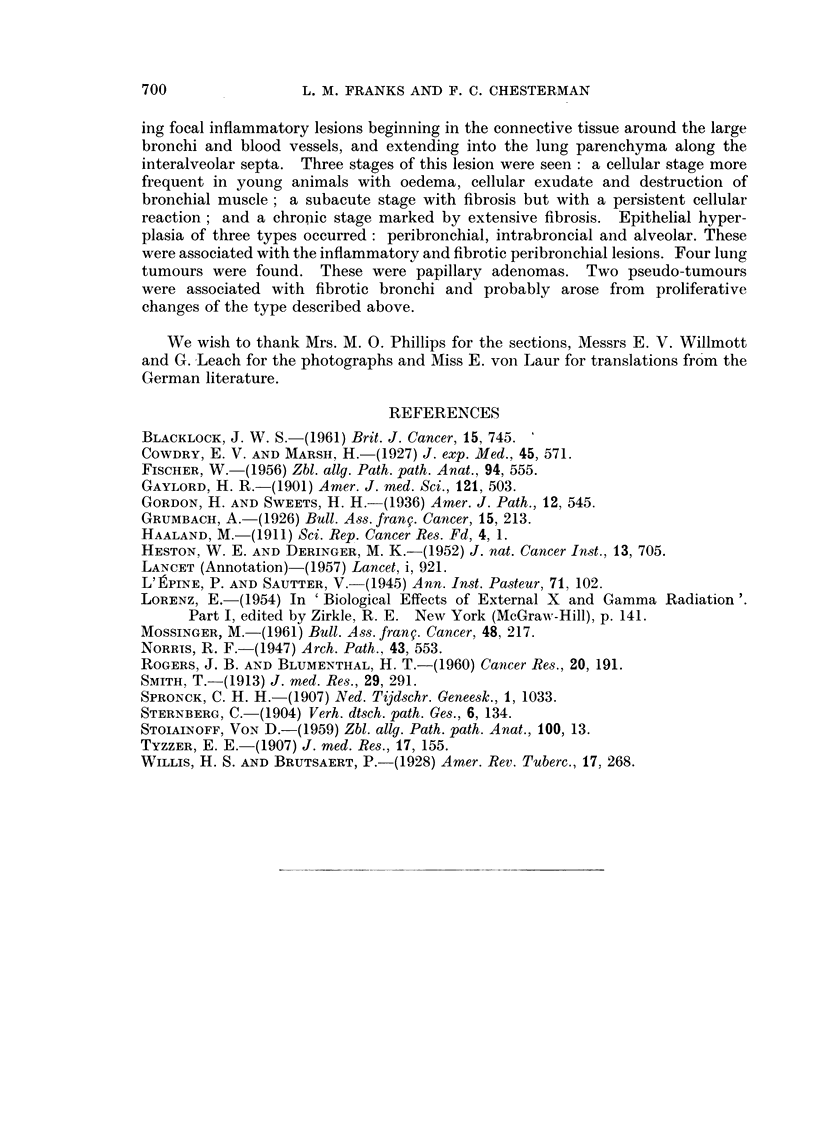# The Pathology of Tumours and other Lesions of the Guinea-Pig Lung

**DOI:** 10.1038/bjc.1962.80

**Published:** 1962-12

**Authors:** L. M. Franks, F. C. Chesterman

## Abstract

**Images:**


### THE PATHOLOGY OF TUMOURS AND OTHER LESIONS OF THE GUINEA-PIG LUNG

L. M. FRANKS AND F. C. CHESTERMAN

*From the Imperial Cancer Research Fund, London, W.C.2 and N.W.7*

Received for publication October 15, 1962

TUMOUR-LIKE nodules in the lungs of guinea-pigs have been reported by several workers. Gaylord (1901) in a paper called " The Protozoan of Cancer " described a nodule of this type after the injection of ascitic fluid from a patient with peritoneal carcinomatosis ; Sternberg (1904) attributed a similar structure to an abnormal bronchial branching and Spronck (1907) reported on a collected series of 56 which he described as spontaneous papillary adenomas of the bronchus. Grumbach (1926) found them in animals inoculated with a corynebacterium isolated from lymph nodes of a patient with Hodgkin's disease, Willis and Brutsaert (1928) in animals exposed to silica dust, and Norris (1947) in a guinea-pig which had been injected with pleural fluid from a patient with lobar pneumonia. These lesions are generally associated with areas of pulmonary fibrosis and in the present paper their incidence, site of origin and possible cause are described. In addition a number of papillary lung tumours are also described. These are uncommon. Heston and Deringer (1952) reported a single tumour, Lorenz *et al.* (1954) 3 and Rogers and Blumenthal (1960) 1. Mossinger (1961), in a recent review, refers to a further 6. Since the tumours we describe were found in old animals the apparent rarity may be due to the fact that relatively few guinea-pigs are allowed to survive till old age.

### MATERIAL AND METHODS

Autopsies were performed on 1080 guinea-pigs (855 males, 225 females) from birth to over 5 years of age. Lung sections were made from 255 of these (220 males, 35 females). One hundred and six were untreated ; 12 were used in transplantation experiments and the remainder were given some form of endocrine treatment (castration, stilboestrol, etc.). Paraffin sections were stained with Ehrlich's haematoxylin and eosin and some were also stained by a combined alcian blue, periodic acid-Schiff, orange G and haematoxylin technique. A reticulin stain (Gordon and Sweets, 1936) was used in some cases.

### RESULTS

The changes in the lungs were similar in treated and untreated males and females. Only 14 guinea-pigs had normal lungs ; most of these were less than 6 months old. Fourteen had bronchopneumonia and 35 had lung abscesses. Most of the animals also had focal inflammatory lesions, often associated with epithelial hyperplasia and in addition 6 tumour-like nodules were found—2 pseudo-tumours and 4 papillary adenomas. Only 2 of the adenomas were noted at autopsy.

*Focal inflammatory lesions*

These lesions began in the connective tissue around the large bronchi and blood vessels, spreading into the lung in the interalveolar septa. The lung lesions were often segmental and involved the area supplied by an affected bronchus. Three stages of these lesions were seen, cellular, subacute and chronic. In the cellular stage (Fig. 1) there was oedema and a cellular exudate of plasma cells and macrophages, and sometimes giant cells, particularly in the interalveolar septa (Fig. 2). There was destruction of bronchial muscle, followed by progressive peribronchial fibrosis. In the subacute stage this was accompanied by a persistent cellular reaction in the bronchial wall and around groups of small acini in the adjacent lung (Fig. 3 and 4). In the chronic stage there was extensive fibrosis confined to the peribronchial and perivascular tissues and rarely affecting the lung substance itself although the alveolar septa were thickened. Many of the affected bronchi were almost completely obstructed either by subepithelial fibrosis or disruption of the whole bronchial wall (Fig. 5). The large blood vessels in the affected areas sometimes showed thickening of the muscle coat and occasionally luminal obstruction due to intimal thickening. Vascular changes were uncommon.

*Epithelial changes*

Epithelial hyperplasia of 3 types occurred : peribronchial, intrabronchial and alveolar. The incidence of these lesions increased with age. The epithelial changes were multifocal and associated with the peribronchial inflammatory and fibrotic lesions.

*Peribronchial hyperplasia.*—In this condition groups of small acini were seen around the periphery of an affected bronchus lying outside the bronchial muscle and surrounding fibrous tissue (Fig. 3 and 4). These are small peribronchial pouches communicating with the lumen through a single narrow opening (Fig. 6). In most sections this opening is not seen.

*Intrabronchial hyperplasia.*—This occurred as a broad-based, rather irregular intrabronchial papilloma, growing from one side of the bronchial wall (Fig. 7). Both peribronchial and intrabronchial proliferation were relatively infrequent.

*Alveolar hyperplasia.*—This type of lesion was the one most often found and consisted of epithelial nodules scattered throughout the lung parenchyma. The epithelium was arranged in small acini (Fig. 8) with little stroma. The nodules varied in size, some being microscopic while others were a millimetre or more in diameter. They appeared to arise from bronchial epithelium which had grown downwards into the bronchioles and alveoli. An early stage in this process is shown in Fig. 9. In the centre there is a mass of muscle, perhaps the remains of an obstructed bronchus, above which is a peribronchial epithelial mass. On its right there is a bronchus cut in its length, with a proliferating mass of epithelium lying in a distended airspace.

*Tumours and pseudo-tumours*

Six-tumour-like nodules were found. Four were papillary adenomas similar to that reported by Heston and Deringer (1952). One was transplanted but failed to grow. Three arose in males 44, 51 and 53 months old and the fourth in a 42-month-old female. Two of the animals had been castrated, the third was untreated but had an adrenal tumour. The structure of the lung tumours is shown in

Fig. 10 and 11. The other 2 nodules were found in males 22 and 36 months old; one of these lesions is illustrated in Fig. 12 and 13. They replaced most of the affected lobe. A large bronchus ran into the mass (Fig. 12) which was surrounded by a dense zone of fibrous tissue in which there were several small abscesses. The central portion was made up of closely packed groups of irregular acini, some cystic, lined by mucin-secreting epithelium. In one lesion there were several spicules of bone (Fig. 13). In both cases the bronchi in other parts of the lung showed peribronchial inflammatory lesions and the younger animal also had areas of intrabronchial and alveolar epithelial proliferation.

*Other lesions*

In 4 animals, all less than 1 year old, foreign bodies were found impacted in large bronchi. In 1 this was surrounded by a polymorph exudate but the others showed a giant cell reaction (Fig. 14), peribronchial fibrosis and epithelial hyperplasia around the involved bronchi. The foreign bodies were identified as being of vegetable origin by Mr. J. Jackson of the Royal Veterinary College. Some had a leaf structure and others resembled seed husks.

*Lung abscesses* were present in 35 animals. Most of these were of inhalation type and sometimes fragments of vegetable material could be found in the abscess cavities (Fig. 15). At the edge of these abscesses there were often areas of epithelial proliferation (Fig. 16).

*Nodules of bone* were present in 15 animals—all male—and all but 1 over 2 years old.

*Peribronchial and alveolar inflammatory lesions*

These are presumably due to some lesion which affects almost all the animals while they are still very young but the cause is unknown. It is unlikely that a primary virus (L'Épine and Sautter, 1945) or bacterial (Smith, 1913) infection

---

EXPLANATION OF PLATES

All sections are stained with Ehrlich's haematoxylin and eosin.

FIG. 1.—Oedema and cellular inflammation of bronchial wall. ×70.
FIG. 2.—Giant cells and macrophages in alveolar septum. ×340.
FIG. 3 and 4.—Peribronchial fibrosis with persistent cellular reaction in the bronchial wall and around groups of small acini in the surrounding lung. Fig. 3 ×70. Fig. 4 ×170.
FIG. 5.—Fibrosis and disruption of bronchial wall. ×70.
FIG. 6.—Peribronchial lesion—groups of small acini (top) outside the wall of a large bronchus. These are small peribronchial pouches communicating with the bronchial lumen through a narrow opening. ×165.
FIG. 7.—Intrabronchial papillary lesion. ×35.
FIG. 8.—Small alveolar epithelial nodule. ×35.
FIG. 9.—There is a mass of muscle in the centre above which is a peribronchial epithelial nodule. On the right there is a bronchus cut in its length with a proliferating mass of epithelium lying in a distended airspace. ×35.
FIG. 10 and 11.—Papillary lung tumour. Fig. 10 ×30. Fig. 11 ×300.
FIG. 12 and 13.—" Pseudo-tumour ". A large bronchus runs into a mass which is surrounded by fibrous tissue in which there are several abscesses. The rest of the nodule consists of closely packed acini lined by mucus-secreting epithelium. Fig. 12 ×9. Fig. 13 ×60.
FIG. 14.—Giant cell reaction around foreign body in a space lined by bronchial epithelium. ×320.
FIG. 15.—Vegetable material in a bronchus at the edge of a lung abscess. ×320.
FIG. 16.—Epithelial proliferation around a lung abscess. ×65.

1

2

3

4

5

6

7

8

9

10

11

12

13

Franks and Chesterman.

14

15

16

would produce such localised changes. Since in some cases vegetable matter, presumably derived from feed or bedding, has been seen impacted in an involved bronchus it is possible that a reaction to this may be the primary cause of the lung lesions.

*Epithelial changes*

The peribronchial, intrabronchial and alveolar epithelial hyperplasia are probably all part of the same process, the type of proliferation depending on the stage of the accompanying inflammatory and fibrotic changes. If the epithelial growth stimulation occurs during the phase of acute or subacute bronchial inflammation, the proliferating epithelium may grow through the damaged bronchial wall and develop into the peribronchial pouches. Willis and Brutsaert (1928) have shown in wax reconstructions that these lesions are groups of branching finger-like protrusions arising from a single narrow main stem which communicates directly with the bronchus. In the " acute " and subacute stages of the inflammatory lesions, small protrusions of epithelium through the damaged muscle are seen frequently and these presumably later grow to peribronchial pouches. If the epithelial growth occurs in the fibrotic stage the cells may grow into and along the bronchi forming intrabronchial papillomata, or alveolar nodules if the epithelium extends into the distal airspaces. However, it is possible that there may also be epithelialisation of the alveoli *in situ* in these cases.

The cause of the epithelial growth stimulation is not known. Tyzzer (1907) and Haaland (1911) have both reported adenomas of lung in mice in which nematode worms were found but we have been unable to identify worms in our material. Willis and Brutsaert (1928) found epithelial nodules in guinea-pigs after dust inhalation and Blacklock (1961) found inflammatory and fibrotic lesions followed by epithelial hyperplasia after the injection of cigarette smoke condensate into the lung. Since we have found epithelialisation around abscesses and in association with vegetable matter it is possible that a number of different stimuli may produce this effect in guinea-pigs. Another possibility which cannot be excluded is that the epithelial lesions may be a response to a virus infection similar to that causing jagziekte in sheep (Cowdry and Marsh, 1927) although bronchial lesions are not prominent in this disease. Small areas of epithelial hyperplasia have also been reported in the human lung, particularly in cases of bronchiectasis or chronic abscesses (see Lancet, 1957, for review) but these are generally solid nests of cells which do not resemble the nodules we have found in the guinea-pigs.

*Lung tumours*

The 4 papillary lung tumours resemble those reported by Heston and Deringer (1952), Rogers and Blumenthal (1960) and Lorenz *et al.* (1954). The other epithelial lesions we have described are similar to those reported by Spronck (1907) and Fischer (1956). Many of these have been included in recent reviews on guinea-pig tumours (Rogers and Blumenthal, 1960 ; Mossinger, 1961) but, as has been suggested by Stoiainoff (1959), we feel these should not be so classified.

### SUMMARY

The lungs of 220 male and 35 female guinea-pigs ranging in age from birth to over 5 years were examined. The lungs were normal in only 14, the others showing focal inflammatory lesions beginning in the connective tissue around the large bronchi and blood vessels, and extending into the lung parenchyma along the interalveolar septa. Three stages of this lesion were seen : a cellular stage more frequent in young animals with oedema, cellular exudate and destruction of bronchial muscle ; a subacute stage with fibrosis but with a persistent cellular reaction ; and a chronic stage marked by extensive fibrosis. Epithelial hyperplasia of three types occurred : peribronchial, intrabroncial and alveolar. These were associated with the inflammatory and fibrotic peribronchial lesions. Four lung tumours were found. These were papillary adenomas. Two pseudo-tumours were associated with fibrotic bronchi and probably arose from proliferative changes of the type described above.

We wish to thank Mrs. M. O. Phillips for the sections, Messrs E. V. Willmott and G. Leach for the photographs and Miss E. von Laur for translations from the German literature.

### REFERENCES

BLACKLOCK, J. W. S.—(1961) *Brit. J. Cancer*, **15**, 745.
COWDRY, E. V. AND MARSH, H.—(1927) *J. exp. Med.*, **45**, 571.
FISCHER, W.—(1956) *Zbl. allg. Path. path. Anat.*, **94**, 555.
GAYLORD, H. R.—(1901) *Amer. J. med. Sci.*, **121**, 503.
GORDON, H. AND SWEETS, H. H.—(1936) *Amer. J. Path.*, **12**, 545.
GRUMBACH, A.—(1926) *Bull. Ass. franç. Cancer*, **15**, 213.
HAALAND, M.—(1911) *Sci. Rep. Cancer Res. Fd*, **4**, 1.
HESTON, W. E. AND DERINGER, M. K.—(1952) *J. nat. Cancer Inst.*, **13**, 705.
LANCET (Annotation)—(1957) *Lancet*, i, 921.
L'ÉPINE, P. AND SAUTTER, V.—(1945) *Ann. Inst. Pasteur*, **71**, 102.
LORENZ, E.—(1954) In ' Biological Effects of External X and Gamma Radiation '. Part I, edited by Zirkle, R. E. New York (McGraw-Hill), p. 141.
MOSSINGER, M.—(1961) *Bull. Ass. franç. Cancer*, **48**, 217.
NORRIS, R. F.—(1947) *Arch. Path.*, **43**, 553.
ROGERS, J. B. AND BLUMENTHAL, H. T.—(1960) *Cancer Res.*, **20**, **191**.
SMITH, T.—(1913) *J. med. Res.*, **29**, 291.
SPRONCK, C. H. H.—(1907) *Ned. Tijdschr. Geneesk.*, **1**, 1033.
STERNBERG, C.—(1904) *Verh. dtsch. path. Ges.*, **6**, 134.
STOIAINOFF, VON D.—(1959) *Zbl. allg. Path. path. Anat.*, **100**, 13.
TYZZER, E. E.—(1907) *J. med. Res.*, **17**, 155.
WILLIS, H. S. AND BRUTSAERT, P.—(1928) *Amer. Rev. Tuberc.*, **17**, 268.